# Ecological Momentary Assessment of the Relationship between Positive Outcome Expectancies and Gambling Behaviour

**DOI:** 10.3390/jcm10081709

**Published:** 2021-04-15

**Authors:** Nicki A. Dowling, Stephanie S. Merkouris, Kimberley Spence

**Affiliations:** 1 School of Psychology, Deakin University, Geelong, VIC 3220, Australia; stephanie.merkouris@deakin.edu.au (S.S.M.); spencek@deakin.edu.au (K.S.); 2 Melbourne Graduate School of Education, University of Melbourne, Parkville, VIC 3053, Australia; 3 School of Psychology, Faculty of Health, Melbourne Burwood Campus, Deakin University, 221 Burwood Highway, Burwood, VIC 3125, Australia

**Keywords:** gambling, outcome expectancies, expenditure, relapse, smartphone, ecological momentary assessment (EMA)

## Abstract

Relapse prevention models suggest that positive outcome expectancies can constitute situational determinants of relapse episodes that interact with other factors to determine the likelihood of relapse. The primary aims were to examine reciprocal relationships between situational positive gambling outcome expectancies and gambling behaviour and moderators of these relationships. An online survey and a 28 day Ecological Momentary Assessment (EMA) were administered to 109 past-month gamblers (84% with gambling problems). EMA measures included outcome expectancies (enjoyment/arousal, self-enhancement, money), self-efficacy, craving, negative emotional state, interpersonal conflict, social pressure, positive emotional state, financial pressures, and gambling behaviour (episodes, expenditure). Pre-EMA measures included problem gambling severity, motives, psychological distress, coping strategies, and outcome expectancies. No reciprocal relationships between EMA outcome expectancies and gambling behaviour (episodes, expenditure) were identified. Moderations predicting gambling episodes revealed: (1) cravings and problem gambling exacerbated effects of enjoyment/arousal expectancies; (2) positive emotional state and positive reframing coping exacerbated effects of self-enhancement expectancies; and (3) instrumental social support buffered effects of money expectancies. Positive outcome expectancies therefore constitute situational determinants of gambling behaviour, but only when they interact with other factors. All pre-EMA expectancies predicted problem gambling severity (OR = 1.61–3.25). Real-time interventions addressing gambling outcome expectancies tailored to vulnerable gamblers are required.

## 1. Introduction

The Diagnostic and Statistical Manual of Mental Disorders (Fifth Edition; DSM-5) [1] now classifies Gambling Disorder (pathological gambling) as an addiction and related disorder. In line with public health frameworks that view gambling problems as occurring across a continuum of risk [2], the term problem gambling is often employed to refer to any gambling that results in adverse consequences for gamblers, families, and communities [3]. Estimates of the standardised global past-year prevalence of adult problem gambling range from 0.5 to 7.6%, with an average of 2.3% [4]. Recent Australian national estimates indicate problem gambling rates of 0.4 to 0.6%, with estimates of a further 1.9 to 3.7% displaying moderate-risk gambling and 3.0 to 7.7% displaying low-risk gambling [5,6]. Moreover, problem gambling is associated with a high burden of harm that is comparable to depression and alcohol use disorders [7]. Harms most often occur across financial, relationship, and emotional domains, with smaller proportions of gamblers reporting physical health problems, cultural harm, reduced work or study performance, and criminal activity [8]. Problem gambling is also associated with a range of comorbid mental health issues, including mood and anxiety disorders, alcohol and other drug use disorders, and personality disorders [9,10,11].

### 1.1. The Relapse Prevention Model

Originally developed to explain relapse in substance abuse, the influential social-cognitive relapse prevention model [12] proposes a classification of factors or situations that can precipitate or contribute to gambling relapse. In general, these factors are classified as covert antecedents (e.g., general stress, lifestyle imbalances, rationalisations and craving) and immediate determinants (e.g., high-risk situations, coping skills, outcome expectancies and the abstinence violation effect). The model assumes that lapses are immediately preceded by high-risk situations, broadly defined as any context that confers vulnerability to gambling (e.g., negative emotional states, interpersonal conflict, social pressure, positive emotional states, and non-specific cravings), but that cognitive and behavioural coping responses impact on self-efficacy to determine whether a high-risk situation culminates in a lapse. Moreover, the abstinence violation effect, combined with positive outcome expectancies, increases the probability of relapse.

A reconceptualisation of the relapse prevention model [13] emphasises the non-linear and dynamic interaction between multiple stable and transient risk factors in high-risk situations to determine the likelihood of relapse, including background factors (e.g., dependence, family history, social support, and comorbid psychopathology), physiological states (e.g., physical withdrawal), cognitive processes (e.g., self-efficacy, outcome expectancies, craving, motivation, the abstinence violation effect), affective states, and coping skills. This model proposes that responses in high-risk situations are related to both distal risk factors (stable predispositions that increase vulnerability to lapse) and proximal risk factors (immediate precipitants that actualise the statistical probability of a lapse) operating within both tonic processes and phasic responses. Tonic processes, which indicate chronic vulnerability for relapse, often accumulate and lead to the instigation of a high-risk situation, thereby determining the initial threshold or “set point” for relapse. In contrast, phasic responses are situational cognitive, affective, or physical states that fluctuate across time and contexts and are conceptualised as high-risk situations that can activate lapses. Phasic responses also include momentary coping responses that can decrease the likelihood of an initial lapse. Tonic processes therefore determine who is vulnerable for relapse, while phasic responses are high-risk situations that determine when relapse will occur. The model incorporates feedback loops, whereby there is a reciprocal causation between cognitive processes (self-efficacy, outcome expectancies, craving, motivation), affective states, coping behaviour, and the addictive behaviour. There is considerable empirical support for the relapse prevention model across the addictions [12,13,14,15,16]. In the context of gambling, however, components of the model lack ecologically valid empirical assessments due to a reliance on traditional cross-sectional methodologies that cannot capture phasic precipitants of gambling nor their dynamic interactions with other phasic or tonic precipitants of gambling in real time.

### 1.2. Positive Outcome Expectancies

Positive outcome expectancies play a central role in these relapse prevention models. Gambling outcome expectancies refer to the anticipated outcome one expects to gain as a result of gambling [12,14,17]. It has been argued that expectancies are an association between mental representations in long-term memory that are automatically activated under specific circumstances [18]. Accordingly, the relapse prevention model hypothesises that positive outcome expectancies become particularly salient in high-risk situations, whereby the possible delayed negative consequences of addictive behaviour are ignored or discounted in favour of the anticipation of immediate positive effects [12]. This is consistent with alcohol expectancy theory, in which vicarious and direct experience with drinking and its consequences shape expectancies for alcohol-related outcomes [19,20]. Systematic review evidence generally supports these assertions, although several factors, such as the measurement of alcohol consumption, the target populations studied, the temporal distance between types of outcome expectancies, and environmental context, may impact the observed relationship between expectancies and alcohol consumption [21].

There is now consistent cross-sectional evidence across samples of varying ages, cultures, and settings that there is an association between problem gambling severity and related harms and positive outcome expectancies. Specifically, evidence indicates that problem gambling severity is associated with specific positive outcome expectancies, including enjoyment/arousal or excitement; self-enhancement, positive self-evaluation or ego enhancement; money or material gain; escape, negative affect, or sedating; and social outcome expectancies [22,23,24,25,26,27,28,29,30], as well as global positive outcome expectancies involving a belief that gambling will make one feel better [31,32,33,34,35,36]. There is also limited prospective research that positive outcome expectancies (excitement, escape, and ego enhancement) predict subsequent gambling problems [37]. Moreover, there is recent evidence of clinically and statistically significant reductions in a global measure of positive outcome expectancies following residential treatment [38].

### 1.3. Ecological Momentary Assessment of Positive Outcome Expectancies

This cross-sectional research, which is subject to recall bias, treats gambling outcome expectancies as stable, enduring traits, rather than transient or phasic responses [39]. The reconceptualised relapse prevention model, however, suggests that transient changes in positive outcome expectancies can constitute phasic determinants of relapse episodes that interact with tonic processes to determine the likelihood of relapse [13]. Ecological Momentary Assessment (EMA), which is an event-level longitudinal methodology, overcomes these limitations by repeatedly measuring symptoms, emotions, behaviour, and thoughts in real time and in natural environments [39]. EMA methodologies, which maximise ecological validity and minimise recall bias, are particularly suitable for examining complex antecedent–consumption–consequence patterns [40,41].

Although EMA methodologies are increasingly being employed in the gambling field [42,43,44,45,46,47,48,49], none have yet examined the relationships between situational (phasic) outcome expectancies and gambling behaviour. This is in contrast to other addictions literature, in which there is EMA evidence suggesting that positive outcome expectancies predict the occurrence and amount of smoking and alcohol consumption [50,51,52,53], moderate the relationships between other situational factors and the likelihood of drinking [53], play a mediating role between other situational factors and smoking/alcohol consumption [54,55,56,57], are associated with smoking self-efficacy and craving [58], and reduce non-smoking intentions [58].

### 1.4. The Current Study

An enhanced understanding of these in-the-moment relationships using EMA methodologies, as well as which gamblers are most vulnerable to outcome expectancies, have implications for the development of tailored interventions targeting positive outcome expectancies for preventing gambling-related harm. The primary aims of this study were therefore to: (1) examine the reciprocal relationships between phasic (EMA-measured) positive gambling outcome expectancies and gambling behaviour (episodes, expenditure) and (2) the degree to which phasic responses (EMA-measured self-efficacy, craving, negative emotional state, interpersonal conflict, social pressure, positive emotional state, and financial pressures) and tonic processes (pre-EMA-measured problem gambling severity, gambling motives, psychological distress, and coping strategies) implicated in the relapse prevention models [12,13] moderate the relationships between phasic gambling outcome expectancies and gambling episodes. Secondary aims were to explore the concordance between phasic (EMA-measured) and tonic (pre-EMA-measured) gambling outcome expectancies; and the degree to which tonic (pre-EMA-measured) positive gambling expectancies predict problem gambling severity.

## 2. Materials and Methods

### 2.1. Participants

This convenience sample consisted of 109 adult, past-month gamblers (39 men, 1 unspecified sex) recruited from the Australian community. Participants were aged between 18 and 55 years (*M* = 28.11, *SD* = 7.77). The majority were Australian born (76.15%), had completed an undergraduate or vocational/trade qualification (62.39%), and were in paid full- or part-time employment (55.96%). The majority of participants had gambled on electronic gaming machines (EGMs) in the past 12 months (66.97%), followed by lotteries (64.22%) and instant scratch tickets (60.55%). Descriptive statistics for this sample are displayed in Table 1.

### 2.2. Measures

#### 2.2.1. Pre-EMA Online Questionnaire

Pre-EMA assessments were conducted via an online questionnaire administered via Qualtrics, which measured demographic and background characteristics (sex, age, country of birth, education, employment and past-year gambling frequency), as well as gambling and mental health variables. These measures were selected for their brevity and good psychometric properties, including high classification accuracy where appropriate [3,59,60,61,62].

**Problem gambling severity.** Past-year problem gambling severity was assessed using the 9-item Problem Gambling Severity Index [PGSI; 3]. Items are rated on a 4-point scale, with response options ranging from (0) never to (3) almost always. Total scores range from 0 to 27, which can be classified into non-problem (score of 0), low-risk (scores of 1–2), moderate-risk (scores of 3–7), and problem (scores of 8–27) gambling [3]. The PGSI has demonstrated excellent internal consistency, validity, sensitivity and specificity in previous research [3].

**Gambling motives**. Gambling motives were measured using the 16-item Gambling Motives Questionnaire-Financial [GMQ-F; [62]]. The GMQ-F measures the frequency of gambling for reasons representing enhancement, social, coping, and financial motives. Each item is scored on a 4-point scale from (1) never or almost never to (4) almost always or always, with subscale scores ranging from 4 to 16. The GMQ-F subscales have demonstrated good internal consistencies (α = 0.64–0.84), as well as construct and discriminant validity [62,63,64].

**Psychological distress.** Past-month psychological distress was assessed via the 6-item Kessler 6 Psychological Distress Scale [K6; [61]]. Based on scoring employed for Australian norms, items are rated on a 5-point scale, ranging from (1) none of the time to (5) all of the time. Total scores range from 6 to 30, which can be classified into low (scores of 6–13), moderate (score of 14–18), high (score of 19–24), or very high risk (score of 25–30). The K6 has demonstrated has demonstrated excellent internal consistency (α = 0.89) [61].

**Coping styles.** Coping styles were assessed using the following 2-item subscales from the Brief-COPE [B-COPE; [59]]: active, planning, positive reframing, emotional support, and instrumental support. Items are scored on a 4-point scale ranging from (1) do not do this at all to (4) do this a lot, with subscale scores ranging from 2 to 8. These subscales have demonstrated adequate to good internal consistency (α = 0.64–0.73) [59].

**Gambling outcome expectancies.** Gambling outcome expectancies were assessed using the 23-item Gambling Expectancies Questionnaire [GEQ: [60]]. The GEQ includes three positive expectancy subscales: enjoyment/arousal (8 items; enjoyment, excitement, and social opportunities from gambling), self-enhancement (4 items; gambling as an opportunity to feel good about oneself, either by impressing peers or establishing autonomy from others), and money (3 items; financial gain from gambling). Items are rated on a 7-point scale ranging from (1) no chance to (7) certain to happen, with subscale scores ranging from 1 to 7. The GEQ positive outcome expectancy subscales have demonstrated good to high internal consistency (α = 0.78–0.86) [60].

#### 2.2.2. EMA

An EMA protocol was developed using a smartphone application (Instant Survey). This protocol employed both time-based sampling (i.e., semi-randomly prompting of individuals to input information about their internal states and ecological contexts) and event-based sampling (i.e., collecting data around a specific, discrete event, which in this case is a gambling episode). During a 28 day period, participants were prompted via push notifications to complete a time-based EMA (t-EMA) delivered through the app at random times during two pre-specified periods each day: morning (9:00 a.m.–12 p.m.) and evening (5:30–8:30 p.m.). Each t-EMA took approximately 1–2 min to complete. Each t-EMA included single items measuring positive outcome expectancies, self-efficacy, craving, negative emotional state, interpersonal conflict, social pressure, positive emotional state, and financial pressures.

Given the lack of validated single items measuring the constructs identified in the relapse prevention models, we selected single items from longer, validated instruments that are well established in the literature, either on the basis of their factor loadings or their representation of constructs from the models. Positive outcome expectancies were measured using the item with the highest factor loading for each GEQ subscale [60]: feel excited (enjoyment/arousal), feel powerful (self-enhancement), and win money (money). Participants rated the likelihood of each outcome expectancy on a 4-point scale, ranging from (0) very unlikely to (3) very likely. Gambling self-efficacy was assessed using the single self-efficacy item from the readiness rulers [65]. Participants rated their confidence in their ability to resist an urge to gamble on a 4-point scale, ranging from (0) strongly disagree to (3) strongly agree. The remaining EMA items employed in this study (craving, negative emotional state, interpersonal conflict, social pressure, positive emotional state, and financial pressures) were measured using items adapted from the from the Inventory of Gambling Situations—Short Form [66]. Each of these items was measured using a single item rated on a 4-point scale, ranging from (0) strongly disagree to (3) strongly agree.

Finally, participants reporting a gambling episode within any t-EMA were administered an event-based EMA (e-EMA) measuring the occurrence of a gambling episode since the previous t-EMA, as well as gambling expenditure. Table 2 presents the EMA items employed, as well as the categories used for the data analysis.

### 2.3. Procedure

Participants were recruited via convenience and snowball sampling using public advertisements, social media platforms, and online groups. Gamblers across the spectrum of risk were recruited given evidence that most of the burden of harm is attributable to low- and moderate-risk gambling [7]. Eligibility criteria included being at least 18 years of age, owning a smartphone, and reporting having gambled in the past-month. Participants completed a 25 min pre-EMA online questionnaire, which confirmed eligibility via self-report questions, gained informed consent, completed pre-EMA measures, and received EMA instructions. This was followed by the 28 day EMA protocol. Of the 373 eligible participants who provided informed consent and completed the pre-EMA questionnaire, only 126 provided valid EMA IDs that could be used to link their pre-EMA and EMA data. Of these 126 participants, 17 did not complete any t-EMAs. The final sample therefore consisted of 109 participants (29.22%) who were compensated with an AUD$20 e-gift voucher. Data was collected from May 2018 to July 2019. Ethics approval was obtained from the Deakin University Human Research Ethics Committee (2018-049).

### 2.4. Data Analysis

All data analyses were conducted using Stata 16 [67]. There was no missing data within the pre-EMA survey due the use of forced-choice responses. Due to skewed distributions, clinical cut-off scores were employed when possible: PGSI (8+: non-problem gambling, problem gambling) and K6 (19+: low or moderate risk, high or very high risk). The remaining pre-EMA measures were dichotomised ad hoc based on response labelling. Specifically, given GMQ-F subscales are derived based on the mean of the relevant items, subscale scores were dichotomised to reflect on average whether participants had ‘never or almost never’ endorsed such an expectancy (mean scores of <2) or had endorsed such an expectancy ‘sometimes/often/always or almost always’ (mean scores of 2+). B-COPE subscales, however, are calculated by summing the relevant items. Subscale scores were therefore dichotomised to reflect whether participants had used each coping style ‘a medium amount/a lot’ for at least one item within the relevant subscale (scores of 5+), with scores of less than 5 reflecting that participants had used the relevant coping style not at all or a little bit across both subscale items (i.e., ‘do not do this at all/do this a little bit). EMA outcome expectancies (‘very unlikely/unlikely’, ‘likely/very likely’), gambling expenditure (‘AUD$0-50′, ‘AUD$51+’), and all remaining EMA items (‘strongly disagree/disagree’, ‘agree/strongly agree’) were also dichotomised.

A series of mixed-effects binary logistic regressions with logit-links examined the reciprocal relationships between EMA outcome expectancies and gambling behaviour (episodes, expenditure). The magnitude of odds ratios (OR) for these main effects were interpreted according to established guidelines: small (OR = 1.68), medium (OR = 3.47), and large (OR = 6.71) [68]. These analyses were repeated to explore moderation effects on the relationship between EMA outcome expectancies and gambling episodes using the relevant interaction term. In all of these analyses, EMA-measured independent and moderator variables were time-lagged to represent participant scores at the t-EMA immediately prior to the outcome of interest, and controlled for age, sex, time, and the outcome measured at the previous t-EMA. Significant interaction effects were explored using pairwise comparisons for marginal means. A series of Spearman’s correlations were employed to examine the concordance between each pre-EMA GEQ item/subscale and its corresponding EMA item, which were interpreted according to established guidelines: negligible (r_s_ = ±0.00 to ±0.30), low (r_s_ = ±0.30 to ±0.50), moderate (r_s_ = ±0.50 to ±0.70), high (r_s_ = ±0.70 to ±0.90), and very high (r_s_ = ±0.90 to ±1.00) [69]. Finally, a series of ordinal univariate logistic regressions and an ordinal multivariate regression explored the degree to which pre-EMA GEQ positive outcome expectancy subscale scores predicted pre-EMA PGSI problem gambling severity categories.

Significance was set at α = 0.05 for the primary analyses investigating the main effects relationships and any pairwise comparisons. However, a more conservative approach towards interpretation of moderation effects was employed due to the large number of moderators and potential for Type 1 error. Specifically, the manuscript presents only those moderation effects for which *p* < 0.03. While seemingly arbitrary, this threshold was considered to provide a reasonable balance between avoiding Type 1 error and Type 2 error, given the small number of participants in between-subjects analyses and the exploratory nature of these analyses. This approach has been employed by the research team in previous studies employing moderation analyses [46,70].

## 3. Results

### 3.1. Descriptive Statistics

Presented in Table 3 are the descriptive statistics for the key pre-EMA variables, broken down by sex. Overall, 91 participants (83.49%) reported gambling problems across the continuum of risk: 25 participants (22.94%) were classified in the PGSI problem gambling category, 42 participants (38.53%) were classified in the PGSI moderate-risk gambling category, and 24 participants (22.02%) were classified in the PGSI low-risk gambling category. Sex differences on key variables were identified for BCOPE emotional support, with more women endorsing greater use of BCOPE emotional support, compared to men. There were no sex differences on PGSI problem gambling severity, GEQ gambling outcome expectancies, GMQ-F gambling motives, or K6 psychological distress. All pre-EMA scales displayed good internal consistency (α = 0.71–0.95). Overall, 3142 t-EMAs were completed (compliance rate = 51.47%), with participants completing a mean of 28.83 t-EMAs (*SD* = 22.07, range = 1–61). Within these t-EMAs, 381 gambling episodes were recorded (*M* = 3.50, *SD* = 6.47, range = 0–38).

### 3.2. Reciprocal Relationships between EMA Gambling Outcome Expectancies and EMA Gambling Behaviour

Presented in Table 4 are the results of the mixed-effects binary logistic regressions. After adjustment for covariates and the outcome at the previous t-EMA, no significant regression effects were identified.

### 3.3. Moderators of the Reciprocal Relationships between EMA Gambling Outcome Expectancies and EMA Gambling Episodes

A series of moderated mixed-effects binary logistic regressions (Table 5) revealed that there were significant interactions (*p* < 0.03) in predicting gambling episodes between: (a) EMA enjoyment/arousal expectancies and EMA cravings, PGSI problem gambling severity, and BCOPE planning; (b) EMA self-enhancement expectancies and EMA positive emotional state and BCOPE positive reframing; and (c) EMA money expectancies and BCOPE emotional support and BCOPE instrumental support.

**Figure 1:**
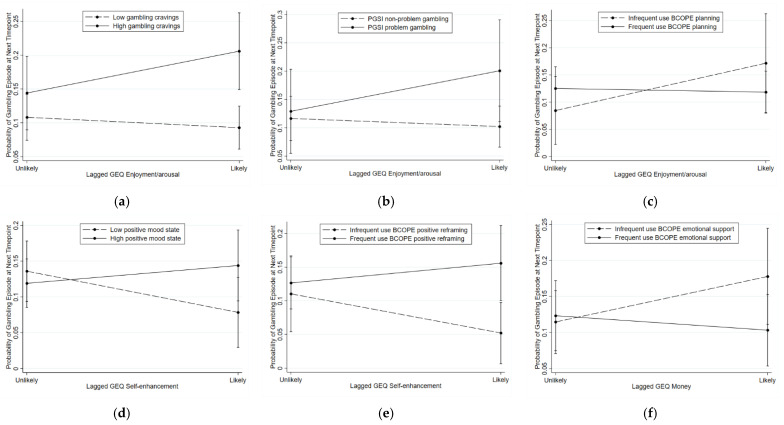

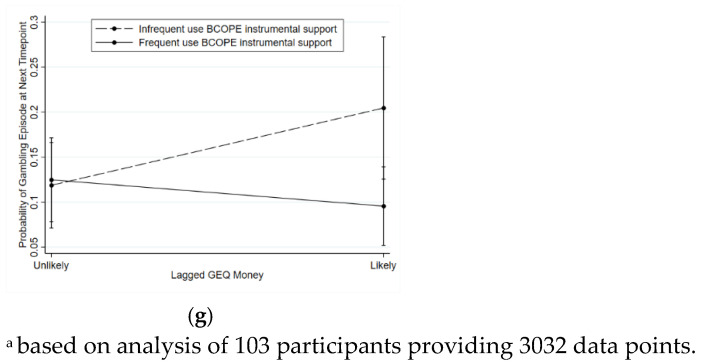
Significant (*p* < 0.03) interaction effects on reciprocal relationships between EMA gambling outcome expectances and gambling episodes ^a^. (**a**) Participants who reported experiencing high cravings (vs. low cravings) had a higher probability of reporting a subsequent gambling episode if they endorsed enjoyment/arousal expectancies (*p* < 0.001). There was no difference between participants who reported experiencing an urge (vs. no urge) if they did not endorse enjoyment/arousal expectancies (*p* = 0.123). (**b**) Participants who reported problem gambling (vs. non-problem gambling) had a higher probability of reporting a subsequent gambling episode if they endorsed enjoyment/arousal expectancies (*p* = 0.022). There was no difference between participants who reported problem gambling (vs. non-problem gambling) if they did not endorse enjoyment/arousal expectancies (*p* = 0.758). (**c**) There were no statistically significant differences between participants who reported frequent (vs. infrequent) use of planning coping on reporting a subsequent gambling episode at either level of enjoyment/arousal expectancies (unlikely: *p* = 0.311; likely: *p* = 0.241). (**d**) Participants who reported experiencing a high positive emotional state (vs. low positive emotional state) had a higher probability of reporting a subsequent gambling episode if they endorsed self-enhancement expectancies (*p* = 0.034). There was no difference between participants who reported experiencing a high positive mood state (vs. low positive mood state) if they did not endorse self-enhancement expectancies (*p* = 0.290). (**e**) Participants who reported frequent use of positive reframing coping (vs. infrequent use) had a higher probability of reporting a subsequent gambling episode if they endorsed self-enhancement expectancies (*p* = 0.012). There was no difference between participants who reported frequent (vs. infrequent) use of positive reframing if they did not endorse self-enhancement expectancies (*p* = 0.638). (**f**) There were no statistically significant differences between participants who reported frequent (vs. infrequent) use of emotional support coping on reporting a subsequent gambling episode at either level of money expectancies (unlikely: *p* = 0.786; likely: *p* = 0.071). (**g**) Participants who reported frequent use of instrumental support (vs. infrequent use) had a lower probability of reporting a subsequent gambling episode if they endorsed money expectancies (*p* = 0.011). There was no difference between participants who reported frequent (vs. infrequent) use of instrumental support if they did not endorse money expectancies (*p* = 0.853).

Pairwise comparisons for these significant interaction effects revealed that: (a) participants who reported experiencing high cravings had a higher probability of reporting a subsequent gambling episode if they endorsed enjoyment/arousal expectancies (Figure 1a); (b) participants who reported problem gambling (vs. non-problem gambling) reported a higher probability of reporting a subsequent gambling episode if they endorsed enjoyment/arousal expectancies (Figure 1b); (c) participants who reported experiencing a high positive emotional state (vs. low positive emotional state) had a higher probability of reporting a subsequent gambling episode if they endorsed self-enhancement expectancies (Figure 1d); (d) participants who reported frequent use of positive reframing as a style of coping (vs. infrequent use) had a higher probability of reporting a subsequent gambling episode if they endorsed self-enhancement expectancies (Figure 1e); and (e) participants who reported frequent use of instrumental support as a style of coping (vs. infrequent use) had a lower probability of reporting a subsequent gambling episode if they endorsed money expectancies (Figure 1g). In these analyses, there were no statistically significant differences between participants who reported frequent use (vs. infrequent use) of planning as a coping style on reporting a subsequent gambling episode at either level of enjoyment/arousal expectancies (Figure 1c) or between participants who reported frequent use (vs. infrequent use) of emotional support as a coping style on reporting a subsequent gambling episode at either level of money expectancies (Figure 1f).

### 3.4. Correlations between Pre-EMA and EMA Gambling Outcome Expectancies

Each EMA gambling outcome expectancy item was significantly correlated with its corresponding pre-EMA single-item (high range: *r_s_* = 0.94–0.98, *p* < 0.001), as well as its corresponding pre-EMA GEQ subscale score (high range: *r_s_* = 0.71–0.73, *p* < 0.001; with the exception of self-enhancement (moderate range: *r_s_* = 0.59, *p* < 0.001).

### 3.5. Relationship between Pre-EMA Gambling Outcome Expectancies and Pre-EMA Problem Gambling Severity

Table 6 displays the ordinal univariate and multivariate logistic regression analyses exploring the degree to which pre-EMA GEQ subscale scores predicted pre-EMA PGSI problem gambling severity categories. In the univariate analyses, all pre-EMA GEQ subscale scores significantly positively predicted PGSI problem gambling severity categories: enjoyment/arousal (OR = 3.25, *p* < 0.001), self-enhancement (OR = 1.81, *p* < 0.001), and money (OR = 1.61, *p* = 0.002). Only enjoyment/arousal (OR = 2.67, *p* < 0.001) and self-enhancement (OR = 1.49, *p* < 0.018) expectancies, however, were significant independent predictors in the multivariate analysis.

## 4. Discussion

This study is the first to employ an EMA methodology to examine the reciprocal relationships between momentary positive gambling outcome expectancies (enjoyment/arousal, self-enhancement, money) and gambling behaviour (episodes and expenditure). This study also explored the moderating role of other factors implicated in the relapse prevention models: phasic responses (EMA-measured self-efficacy, craving, negative emotional state, interpersonal conflict, social pressure, positive emotional state, and financial pressures) and tonic processes (pre-EMA-measured problem gambling severity, gambling motives, psychological distress, and coping strategies) [12,13]; the concordance between the phasic (EMA-measured) and tonic (pre-EMA-measured) positive outcome expectancies; and the associations between tonic (pre-EMA-measured) positive outcome expectancies and problem gambling severity.

### 4.1. Reciprocal Relationships between EMA Gambling Outcome Expectancies and EMA Gambling Behaviour

Contrary to the primary hypothesis, none of the positive outcome expectancies measured in the EMA were reciprocally related to gambling episodes or gambling expenditure. These findings challenge the relapse prevention models, which emphasise the role of positive outcome expectancies as precipitants to relapse and posit that there are feedback loops between transient changes in positive outcome expectancies and gambling episodes [12,13]. They are also inconsistent with previous EMA substance use research demonstrating that these expectancies are phasic determinants of smoking and alcohol consumption [50,51,52] and previous cross-sectional research demonstrating a significant relationship between positive gambling outcome expectancies and problem gambling [22,23,24,25,26,27,28,29,30].

Several methodological considerations may explain these findings. First, the items selected to represent each gambling expectancy item were measured using the item with the highest factor loading for each GEQ subscale [60]. Although the GEQ consists of three discrete subscales of positive outcome expectancies, it has been acknowledged that the complexity of items identified within the enjoyment/arousal and self-enhancement subscales are not as discrete [60]. Specifically, the enjoyment/arousal subscale includes items denoting enjoyment, excitement, relief from boredom, escape/tension reduction and social interaction, while the self-enhancement scale includes items reflecting potential outcomes of social gains as well as independence. It was argued that these subscales represent new ways of viewing gambling from an adolescent perspective, the sample for which the GEQ was developed. By selecting the highest loading item for each of these subscales, this study therefore appeared to measure the constructs of excitement (for enjoyment/arousal) and independence (for self-enhancement) in the EMA. This is supported by the current study’s findings that the correlations between tonic (pre-EMA) and phasic (EMA) gambling outcome expectancies were significant, yet relatively low compared to what would represent good convergent validity, i.e., r > 0.70 [71]. Moreover, noticeably absent from the GEQ positive expectancy subscales are items representing escape or tension reduction, which is considered an important determinant of gambling problems [60]. Although the GEQ is one of the most commonly used measures of gambling expectancies in gambling research, future research replicating this study using items selected from a measure developed for adult samples with more discrete subscales, such as the more contemporary Gambling Outcome Expectancy Scale (GOES) [22], is required.

Second, the failure to identify significant reciprocal relationships between positive outcome expectancies may be due to the use of a relatively small convenience sample of past-month gamblers. The sample size may have resulted in underpowered analyses involving time invariant factors and wide confidence intervals, particularly for expenditure data, which indicate reduced certainty in the results. The methods of recruitment, including social media platforms, may under-represent more vulnerable and socially excluded individuals, who are particularly vulnerable to developing gambling problems, and resulted in an over-representation of other population subgroups, such as fulltime students. Moreover, the assumptions underpinning the relapse prevention model, which were developed to explain relapse behaviour in people with dependence, were tested with a sample of past-month gamblers recruited from the community rather than a problem gambling sample. However, the majority (84%) of participants reported gambling problems, which allowed for the use of a problem gambling threshold for moderation analyses. Moreover, problem gambling moderated only one of the observed relationships, suggesting that there were few differences in the magnitude of effects dependent on problem gambling status. It is important to include gamblers across the spectrum of risk when exploring the mechanisms underlying gambling behaviour, given findings that the majority (85%) of the burden of harm associated with gambling problems is attributed to low- and moderate-risk gamblers due to their higher prevalence in the population [7].

Alternatively, it may be that positive outcome expectancies measured as phasic responses do not display reciprocal relationships with gambling behaviour. As the first EMA study to explore gambling outcome expectancies, this study focused on the investigation of the reciprocal relationship between gambling outcome expectancies and gambling behaviour and the moderators of these relationships. As evidenced in the addictions literature [53,54,55,56,57], it may be that positive outcome expectancies either mediate or moderate the relationships between other situational factors and gambling behaviour. Given this emerging field of study, future studies are required to investigate the potential role of positive gambling outcome expectancies as mediators and moderators of the relationships between other precipitating factors implicated in the relapse prevention models and subsequent gambling behaviour.

### 4.2. Moderators of the Reciprocal Relationships between EMA Gambling Outcome Expectancies and EMA Gambling Episodes

While the failure to identify reciprocal relationships between positive outcome expectancies and gambling behaviour may be interpreted to suggest that the relapse prevention models are not as applicable to this behavioural addiction as substance use addictions, there is now evidence using EMA methodologies to suggest that gambling cravings and self-efficacy, which are other central cognitive processes implicated in the relapse prevention models, play a critical role in predicting gambling behaviour [46]. Moreover, the associations between EMA positive outcome expectancies and subsequent gambling episodes were moderated by several phasic responses (EMA cravings, positive emotional state) and tonic processes (pre-EMA PGSI problem gambling, BCOPE positive reframing, BCOPE instrumental support). These findings therefore suggest that transient changes in positive outcome expectancies do constitute phasic determinants of gambling episodes, but only when they interact with these tonic and phasic processes [13].

Specifically, the moderation analyses relating to enjoyment/arousal expectancies indicate that participants who reported experiencing high cravings or problem gambling had a higher probability of reporting a subsequent gambling episode if they endorsed enjoyment/arousal expectancies. These findings suggest that anticipation of enjoyment, excitement, and social opportunities play an important role in predicting the likelihood of gambling for gamblers who are having gambling cravings or who have gambling problems. These findings highlight the importance of developing interventions to reduce these expectancies for problem gamblers, as well as real-time interventions to reduce these expectancies when gamblers are experiencing cravings.

The moderation analyses relating to self-enhancement expectancies indicate that participants who reported experiencing a high positive emotional state or more frequent use of positive reframing as a coping style had a higher probability of reporting a subsequent gambling episode if they endorsed self-enhancement expectancies. The finding relating to high positive emotional state suggests that anticipation that gambling provides an opportunity to feel good about oneself, either by impressing peers or establishing autonomy from others play an important role in predicting the likelihood of gambling for gamblers who report positive mood states. This finding highlights the need for the development of real-time interventions to reduce these expectancies when gamblers report high positive mood. The finding relating to positive framing, however, is somewhat counterintuitive, given that positive reframing is commonly conceptualised as an emotion-focused [72,73] or approach [74] coping style that is an adaptive response to stress [72]. However, as a type of emotion-focused coping, positive reframing aims to manage distressing emotions rather than to deal with the stressor per se [72,73]. Moreover, it has been emphasised that certain coping responses may be beneficial for some people in some situations, but less helpful for other people or in other situations [72]. That is, positive reframing may not be intrinsically maladaptive, but may become dysfunctional in certain contexts. It therefore may be that, in the context of gambling, anticipation of feeling good about oneself plays an important role in predicting the likelihood of gambling for gamblers who tend to find something good in the situation because they minimise the negative consequences of gambling. Given that this construct was measured using a brief instrument administered prior to the EMA, future research that measures positive reframing in the EMA is necessary.

The moderation analyses relating to money expectancies indicate that participants who reported frequent use of instrumental support as a style of coping had a lower probability of reporting a subsequent gambling episode if they endorsed money expectancies. This finding suggests that anticipation of financial gain from gambling plays a less important role in predicting the likelihood of gambling for gamblers who frequently seek advice, assistance, or information. In the context of gambling, instrumental social support therefore appears to be a problem-focused coping style [72] that plays a protective role by buffering the influence of money expectancies, suggesting that it may be important for interventions to enhance instrumental support as a coping style for gamblers who have high money expectancies.

### 4.3. Relationship between PRE-EMA Gambling Outcome Expectancies and Pre-EMA Problem Gambling Severity

Despite the failure to identify relationships between phasic (EMA) gambling outcome expectancies and gambling behaviour, the results revealed that all pre-EMA positive gambling outcome expectancies predicted problem gambling severity. These findings, which are consistent with previous cross-sectional gambling research [22,23,24,25,26,27,28,29,30], suggest that anticipation of positive outcomes from gambling, such as enjoyment, excitement, social opportunities, feeling good about oneself, and financial gain, is associated with the development of higher gambling problems. In the multivariate analyses, however, enjoyment/arousal and self-expectancies independently predicted problem gambling severity, while money expectancies did not. This finding is consistent with previous research in which money expectancies or motives did not contribute to the explanation of gambling problems when non-financial gambling expectancies or motives were taken into account [22,75,76]. These findings suggest that although the chance to win money is central to gambling behaviour, anticipation of winning money is not the main explanation for the development of gambling problems. Instead, it appears that gambling for other reasons, such as gambling for excitement, to feel good about oneself, and to regulate mood, explain continued gambling in the face of increasing losses [22].

There were no sex differences in gambling outcome expectancies in this study, which is not consistent with previous research that has found that males are significantly more likely than females to endorse positive outcome expectancies [23,24,26] and that gender moderates the relationship between some outcome expectancies and problem gambling severity [23,26]. Given that sex differences have implications for the development of gendered treatment approaches, further research clarifying these results is required.

While these findings suggest that positive outcome expectancies are an important risk factor for the development of gambling problems, they are based on cross-sectional associations. Longitudinal research is therefore required to explore the temporal relationship between these expectancies and problem gambling. These findings, however, add to the growing literature that positive gambling outcome expectancies are clear targets for prevention and intervention efforts. Since escape and enhancement expectancies appear to be more important than money expectancies, interventions may benefit by targeting these drivers of gambling problems.

### 4.4. Study Limitations

The study findings should be interpreted in the context of several limitations. As previously mentioned, limitations include the use of a sample of past-month gamblers recruited via convenience and snowball sampling who were required to own a smartphone, although problem, moderate-risk, and low-risk gamblers were over-represented (84% of the sample); an under-representation of males in the sample, who generally display a higher prevalence of gambling problems; a relatively small sample that may have resulted in underpowered moderation analyses and wide confidence intervals, particularly for expenditure data; and relatively low convergent validity of the expectancies EMA item with its corresponding multiple-item GEQ subscale [71]. Other study limitations include low compliance rates (55.31%) compared to other EMA studies [46,77], measuring some constructs (coping and motives) articulated in the relapse prevention model as tonic processes in the pre-EMA survey; and the recording of e-EMAs in t-EMAs, which may have increased recall bias [78]. There were also limitations associated with collecting self-report data, particularly in relation to gambling expenditure [79], although the use of EMA likely increased the accuracy of the gambling expenditure data; as well as a lack of more detailed clinical information relating to psychiatric comorbidities and general health, which have the potential to influence the findings.

Future EMA studies would benefit from incentivising compliance rates, maximising convergent validity using alternative outcome expectancy EMA items, adding EMA items representing escape or tension reduction outcome expectancies, employing time-variant measures of coping and motives in the EMA, and employing user-initiated e-EMAs in large samples of participants with gambling problems. It would also be of interest to explore the potential role of gambling outcome expectancies as moderators or mediators between other situational factors implicated in relapse prevention models, such as affective states, craving, gambling self-efficacy, and gambling motives.

### 4.5. Clinical Implications

Nevertheless, the findings of this study have important clinical implications. They highlight the importance of targeting gambling outcome expectancies in gambling interventions. Psychological strategies derived from the relapse prevention models are generally behavioural or cognitive in therapeutic orientation, but increasingly include third wave approaches, including mindfulness- and acceptance-based strategies [12,13,14,15]. Cognitive-behavioural interventions are considered “best practice” in the treatment of problem gambling [80,81,82], while third-wave strategies are emerging as promising interventions [83,84]. Psychological strategies focusing on positive outcome expectancies within these interventions explore the validity and reality of outcome expectancies by contrasting the possible immediate positive consequences with the delayed negative consequences of gambling [85], as well as other strategies, such as personalised feedback [29]. Meta-analytic evidence supports the efficacy of such expectancy challenge interventions for alcohol abuse prevention [86], as well as personalised feedback interventions for problem gambling [87]. It may also be important for interventions to enhance instrumental support as a coping style for gamblers with high outcome expectancies and examine whether gamblers are inappropriately employing positive reframing as a coping style.

Moreover, they indicate a need for interventions designed to target momentary positive outcome expectancies that change across time and contexts. Just-In-Time Adaptive Interventions (JITAIs) are emerging mobile health (mHealth) intervention designs that address dynamically changing individual needs by providing the type and amount of support, at the right time, and only when needed [88,89]. JITAIs leverage mobile and wireless technologies, such as smartphone-embedded or wearable sensors and EMAs to continuously monitor dynamic internal states and ecological contexts in real time to identify when and how support should be offered [89]. Dynamic and individually tailored JITAIs that employ EMAs as their method of assessment have also been described as Ecological Momentary Interventions (EMIs) [90]. JITAIs and EMIs have been effective in the broader mental health and addiction fields [88,89,90,91]; and have recently been adopted in the gambling field [92,93,94,95,96]. For example, GamblingLess: Curb Your Urge [95,96] is informed by the relapse prevention model and aims to reduce gambling cravings to prevent subsequent gambling episodes. This smartphone-delivered intervention, which was adapted from GamblingLess, an evidence-based online self-directed program for gambling [97,98,99], tailors craving management activities to EMAs evaluating craving intensity; and also provides these activities on-demand. In line with the findings of the current study, such interventions could also be tailored to individuals most vulnerable to gambling in response to transient positive gambling outcome expectancies, such as problem gamblers, and delivered when they need the most support, such as when they are experiencing cravings or positive mood states. Accordingly, under development is a gambling intervention that builds on GamblingLess: Curb Your Urge by adding intervention strategies to reduce positive outcome expectancies and improve self-efficacy in high-risk situations. Delivered via a smartphone app, GamblingLess: In-The-Moment aims to provide tailored interventions to gamblers who report a state of cognitive vulnerability characterised by high craving intensity, low self-efficacy, and positive outcome expectancies in EMAs sent three times a day.

## 5. Conclusions

This EMA study provided important information about the role of positive outcome expectancies as phasic determinants of gambling behaviour. Although there were no reciprocal relationships between EMA-measured positive gambling outcome expectancies and gambling behaviour, several factors implicated in the relapse prevention models (cravings, problem gambling severity, positive emotional states, positive reframing coping, and instrumental social support) moderated the relationships between phasic gambling outcome expectancies and gambling episodes. These findings therefore suggest that transient changes in positive outcome expectancies do constitute phasic determinants of gambling episodes, but only when they interact with these tonic and phasic processes. Moreover, all positive outcome expectancies measured prior to the EMA period predicted problem gambling severity, although money expectancies failed to remain significant after accounting for the other positive outcome expectancies. These findings have important clinical implications, particularly relating to the development of real-time interventions that provide the type and amount of support when and where gamblers need it most.

## Figures and Tables

**Table 1:** Descriptive statistics (*n* = 109).

Variable	*n*	%
Age (*M*, *SD*)	28.11	7.77
Sex		
Male	39	35.78
Female	69	63.30
Other	1	0.92
Born in Australia	83	76.15
Education level		
Post-graduate degree	13	11.93
Undergraduate degree	35	32.11
Vocational or trade certificate	33	30.28
Completed high school	23	21.10
Did not complete high school	5	4.59
Employment status (*n*, %)		
Full-time employment	38	34.86
Part-time employment	23	21.10
Household duties	8	7.34
Full-time student	38	34.86
Unemployed	1	0.92
Unable to work/pension	1	0.92
Past-year gambling participation		
Electronic gaming machines	73	66.97
Horse or greyhound racing	54	49.54
Instant scratch tickets	66	60.55
Lottery	70	64.22
Keno	32	29.36
Casino table games	48	44.04
Bingo	22	20.18
Sporting or other events	50	45.87
Informal private games	30	27.52

**Table 2:** EMA items.

EMA Items	Response Options	Categories Employed in the Analysis
t-EMA Items		
*Positive outcome expectancies*		
How likely do you think these outcomes would be if were to gamble RIGHT NOW? You would…	(0) very unlikely (1) unlikely (2) likely (3) very likely	(0) very unlikely/unlikely (1) likely/very likely
…feel excited		
…feel powerful		
…win money		
*Self-efficacy and high-risk situations*		
Please indicate the degree to which you agree to each of the following statements. RIGHT NOW…	(0) strongly disagree (1) disagree somewhat (2) agree somewhat (3) strongly agree	(0) disagree somewhat/strongly disagree (1) strongly agree/agree somewhat
…I am confident that I would be able to resist the urge to gamble		
…I have an urge to gamble		
…I am having unpleasant or sad or bad feelings		
…I am having difficulties with others		
…I am under social pressure to gamble		
…I am having pleasant or happy or good feelings		
…I am worried about debt		
**e-EMA items**		
*Gambling event*		
Have you gambled since the last time we contacted?	(0) no (1) yes	(0) no (1) yes
*Gambling expenditure*		
Did you win or lose overall?	(1) won (2) lost (3) broke even	(0) won/broke even/lost $1–$50 (1) Lost $51+
How much money did you win or lost in total? If you broke even enter $0	18 categories ranging from $0 to $7501–10,000.	

**Table 3:** Pre-EMA descriptive statistics for key variables.

Variable	Men (*n* = 39)	Women (*n* = 69)	Total (*n* = 109) ^a^	Cronbach’s Alpha
PGSI problem gambling severity (*n*, %)				0.89
Non-problem gambling	3 (7.69)	15 (21.74)	3 (7.69)	
Low-risk gambling	10 (25.64)	14 (20.29)	10 (25.64)	
Moderate-risk gambling	15 (38.46)	26 (37.68)	15 (38.46)	
Problem gambling	11 (28.21)	14 (20.29)	25 (22.94)	
GEQ outcome expectancies (M, SD)				
Enjoyment	4.88 (0.65)	4.68 (1.00)	4.76 (0.89)	0.85
Self-enhancement	2.98 (1.22)	2.75 (1.42)	2.83 (1.35)	0.86
Money	3.75 (1.30)	3.70 (1.17)	3.71 (1.21)	0.82
GMQ-F motives (sometimes/often/almost always or always) (*n*, %)				
Coping	11 (28.21)	19 (27.54)	31 (28.44)	0.91
Enhancement	32 (82.05)	48 (69.57)	81 (74.31)	0.92
Social	19 (48.72)	38 (55.07)	57 (52.29)	0.87
Financial	28 (71.79)	51 (73.91)	80 (73.39)	0.85
K6 psychological distress (high/very high risk) (*n*, %)	6 (15.38)	10 (14.49)	16 (14.68)	0.88
BCOPE coping (do this a medium amount/do this a lot) (*n*, %)				
Active	33 (84.62)	61 (88.41)	95 (87.16)	0.71
Planning	27 (69.23)	58 (84.06)	86 (78.90)	0.84
Positive reframing	32 (82.05)	45 (65.22)	78 (71.56)	0.84
Emotional support	17 (43.59)	44 (63.77)	61 (55.96) *	0.90
Instrumental support	22 (56.41)	47 (68.12)	70 (64.22)	0.95

* *p* < 0.05 for chi-square tests and *t*-tests exploring sex differences in key pre-EMA variables. ^a^ Total sample size including one participant with unspecified sex.

**Table 4:** Mixed effects binary logistic regressions exploring the reciprocal relationships between EMA gambling outcome expectancies and gambling behaviour.

Predictors	Outcomes OR (95%CI)		
	*Gambling episode* ^a^	*Gambling expenditure ^b^*	
*Enjoyment/arousal*	1.12 (0.78, 1.62)	1.61 (0.54, 4.85)	
*Self-enhancement*	1.03 (0.68, 1.57)	0.43 (0.10, 1.76)	
*Money*	1.35 (0.92, 1.98)	1.05 (0.32, 3.46)	
	*Enjoyment/arousal*	*Self-enhancement*	*Money*
*Gambling episode* ^a^	1.14 (0.77, 1.70)	1.02 (0.65, 1.59)	1.16 (0.77, 1.75)
*Gambling expenditure ^b^*	0.35 (0.03, 3.49)	0.37 (0.05, 2.69)	3.54 (0.34, 36.64)

*Note.* OR = odds ratio, CI = 95% confidence interval for OR; all analyses were adjusted for age, sex, and outcome measured at previous time point. ^a^ based on analysis of 103 participants providing 3032 data points; ^b^ based on 64 participants providing 361 observations.

**Table 5:** Moderated regression analyses of the relationships between EMA gambling outcome expectances and gambling episodes ^a^.

Outcome	OR	SE	*p*-Value	95% CI
Enjoyment/arousal × cravings	2.17	0.72	0.019	1.13–4.15
Enjoyment/arousal × PGSI problem gambling	2.42	0.97	0.028	1.10–5.31
Enjoyment/arousal × BCOPE planning	0.32	0.16	0.023	0.12–0.86
Self-enhancement × positive emotional state	2.89	1.27	0.016	1.22–6.86
Self-enhancement × BCOPE positive reframing	3.69	1.95	0.013	1.31–10.37
Money × BCOPE emotional support	0.39	0.16	0.022	0.18–0.88
Money × BCOPE instrumental support	0.29	0.12	0.002	0.13–0.64

^a^ Based on analysis of 103 participants providing 3032 data points.

**Table 6:** Ordinal logistic regression analyses of pre-EMA gambling outcome expectancies predicting PGSI problem gambling severity (*n* = 109).

GEQ Outcome Expectancy Subscale	Univariate Models			Multivariate Model		
	OR	95% CI	*p*	OR	95% CI	*p*
Enjoyment/arousal	3.25	2.01–5.26	<0.001	2.67	1.62–4.42	<0.001
Self-enhancement	1.81	1.36–2.40	<0.001	1.49	1.07–2.08	0.018
Money	1.61	1.19–2.17	0.002	1.11	0.77–1.59	0.583

## Data Availability

The data and code will be available upon request.
